# NA_mCNN: Classification of Sodium Transporters in Membrane Proteins by Integrating Multi-Window Deep Learning and ProtTrans for Their Therapeutic Potential

**DOI:** 10.1021/acs.jproteome.4c00884

**Published:** 2025-04-07

**Authors:** Muhammad Shahid Malik, Van The Le, Yu-Yen Ou

**Affiliations:** † Department of Computer Science and Engineering, Yuan Ze University, Chung-Li 32003, Taiwan; ‡ Department of Computer Sciences, Karakoram International University, Gilgit-Baltistan 15100, Pakistan; § Graduate Program in Biomedical Informatics, Yuan Ze University, Chung-Li 32003, Taiwan

**Keywords:** pretrained protein language models (PLMs), predictive modeling, sodium transporters, protein sequence classification, drug targets

## Abstract

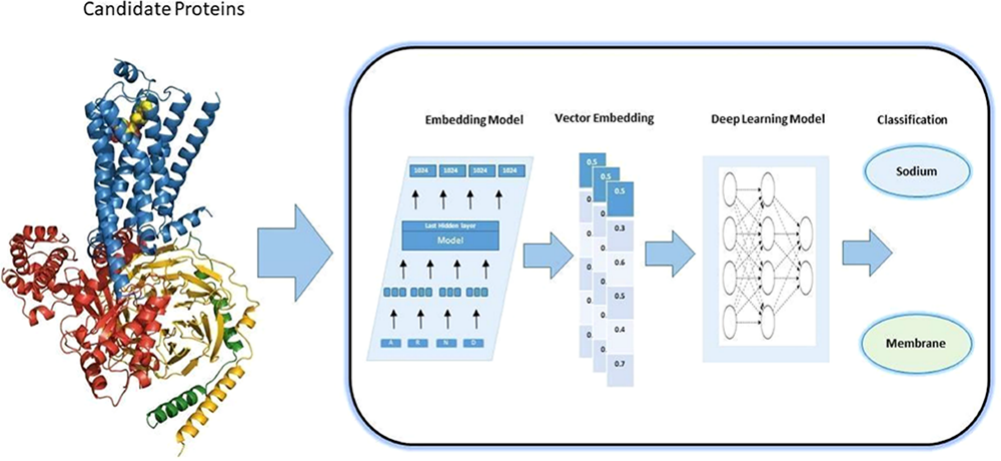

Sodium transporters maintain cellular homeostasis by transporting ions, minerals, and nutrients across the membrane, and Na+/K+ ATPases facilitate the cotransport of solutes in neurons, muscle cells, and epithelial cells. Sodium transporters are important for many physiological processes, and their dysfunction leads to diseases such as hypertension, diabetes, neurological disorders, and cancer. The NA_mCNN computational method highlights the functional diversity and significance of sodium transporters in membrane proteins using protein language model embeddings (PLMs) and multiple-window scanning deep learning models. This work investigates PLMs that include Tape, ProtTrans, ESM-1b-1280, and ESM-2-128 to achieve more accuracy in sodium transporter classification. Five-fold cross-validation and independent testing demonstrate ProtTrans embedding robustness. In cross-validation, ProtTrans achieved an AUC of 0.9939, a sensitivity of 0.9829, and a specificity of 0.9889, demonstrating its ability to distinguish positive and negative samples. In independent testing, ProtTrans maintained a sensitivity of 0.9765, a specificity of 0.9991, and an AUC of 0.9975, which indicates its high level of discrimination. This study advances the understanding of sodium transporter diversity and function, as well as their role in human pathophysiology. Our goal is to use deep learning techniques and protein language models for identifying sodium transporters to accelerate identification and develop new therapeutic interventions.

## 1 Introduction

**Figure 1:**
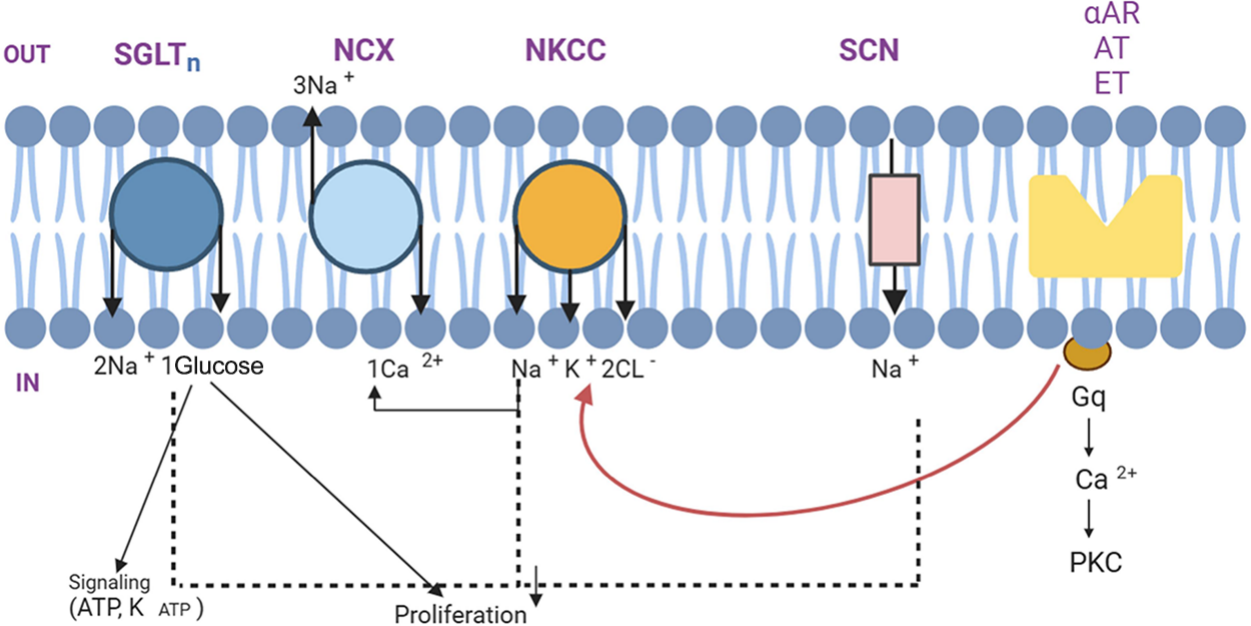
Signaling roles of sodium-dependent transporters. In the diagram, sodium-glucose cotransporters (SGLT), sodium–calcium exchangers (NCX), sodium–potassium–chloride cotransporters (NKCC), and sodium channels (SCN) are illustrated how alpha-adrenergic receptors (aAR) regulate these transporters.

Sodium transporters regulate sodium (Na+) balance across cell membranes, which is vital for a variety of physiological functions. Na+/K+ ATPase pumps facilitate the movement of ions, minerals, and other molecules across cell membranes by establishing electrochemical gradients. Sodium is vital to the uptake of glucose, neurotransmission, calcium signaling, and maintaining acid–base balance within cells. SGLTs (sodium-coupled glucose transporters) and sodium–calcium exchangers (NCX) regulate essential metabolic and cardiovascular functions.^1−4^ The transporter NKCC1 oversees ion homeostasis and cell function while also facilitating nutrients and signaling molecules transport. It has been suggested that NKCC1 mutations may be responsible for Bartter syndrome, a renal disorder associated with electrolyte imbalance.^1,5^ SLC6 family members, such as serotonin and dopamine transporters, illustrate the functional diversity and physiological significance of this class.^6^ These transporters play a crucial role in neural function and neurotransmission by transporting substrates across the cell membrane using the sodium gradient. Many diseases and disorders have been associated with sodium transporter dysfunction or dysregulation, including hypertension, heart failure, kidney disease, and neurological disorders, influencing the treatment of hypertension and heart failure.^7−11^ In addition to NKCC1, sodium-coupled transporters such as SGLTs play pivotal roles in cellular metabolism.^3^ This pathway contributes significantly to glucose homeostasis and serves as a therapeutic target in treating diabetes.^12^ SGLT1 and SGLT2 play a crucial role in renal glucose reabsorption and blood glucose regulation.^13^ These proteins are critical for physiological processes as shown in Figure 1, such as ion balance and maintaining cell volume through their regulation of ion transport across cell membranes. Therefore, it is imperative to understand sodium transporters’ distinct functional properties and disease associations. In addition to maintaining cell balance and preventing associated diseases, sodium transporters play an important role in various health problems, so understanding their function could lead to targeted therapeutic interventions.

The study of sodium transporters provides us with a deeper understanding of cell physiology as well as potentially beneficial pharmacological opportunities across a variety of diseases. Understanding sodium transporter function will allow us to identify novel drug targets and develop targeted therapeutic interventions. SGLT targeting, for example, has shown to be an effective strategy for reducing diabetes symptoms.^14^ SGLT inhibitors, such as canagliflozin and dapagliflozin, have revolutionized diabetes treatment by lowering blood glucose levels and increasing urine glucose excretion.^15^ In addition, sodium transporter dysregulation may offer pharmacological avenues for hypertension and heart failure, among other cardiovascular diseases, as SGLT2 inhibitors have been shown to improve heart failure and reduced ejection fraction (HFrEF) in a significant way.^16^ Understanding sodium transporters’ role in neurological disorders such as Bartter syndrome makes them particularly relevant as therapeutic targets for electrolyte imbalances.^17^ In addition, advances in sodium transporter research have opened the possibility of tailoring therapeutic interventions to the specific needs of individual patients.^18^ There are several inhibitors targeting sodium-dependent transporters currently being investigated as potential drugs to treat hypertension and neurological disorders.^19^ The therapeutic potential of sodium transporters can improve patient outcomes and disease management, but the lack of annotated data and the high degree of sequence similarity between different subtypes complicate the classification of sodium transporters.

Computational methods have revolutionized the classification of transporter proteins. Although sodium transporters have not been specifically studied, several approaches have succeeded in identifying other transporter families, demonstrating the importance of deep learning and machine learning. GT-Finder^20^ uses pretrained BERT language models with Support Vector Machines (SVM) to classify sodium-glucose (SGLT), GLUT, and SWEET transporters. Word embeddings^21^ are also efficient methods of representing protein sequences and identifying substrate specificities in transporter classes, including ions, electrons, and protein/mRNA transporters. In the RBF network-based method,^22^ ion transporter targets are predicted using position-specific scoring matrices (PSSM) and biochemical properties, while in TranCEP,^23^ composition, evolution, and positional data are utilized to predict substrate classes of transmembrane transport proteins with multiclass SVM. In recent years, a hybrid method combining ProtBERT-BFD^24^ with convolutional neural networks has been developed to identify membrane transport proteins. Although these methods advance transporter classification, a notable gap remains in sodium transporter classification.

In recent years, advances in computational biology have revolutionized our understanding of the functional diversity of these transporters and the associations between them and disease.^25^ Through the use of pretrained protein language models (PLMs), as well as advanced predictive modeling tools, scientists have made significant progress in deciphering complex patterns and interactions in bioinformatics. Pretrained language models, due to their versatile applications spanning dialogue systems, text generation, and even computer programming, techniques like BERT^26,27^ and GPT-3^28^ have garnered considerable attention. During the past few years, the field has witnessed a surge of studies advocating for the development of large-scale pretrained language models tailored specifically for protein sequences.^29−31^ ProtTrans,^29^ a model developed by Burkhard Rost and his team, is one example.

Multiple window scanning was introduced for convolutional neural networks (CNNs) to enhance feature extraction, capturing diverse patterns at different scales.^32^ Further, Seo and colleagues^33^ adapted this approach to protein classification, leveraging its ability to detect key motifs across biological sequences. The development of features for membrane protein sequences has revolutionized computational methodologies from multiple sequence alignment to language models based on amino acid sequences to gain insight into protein structure and function, especially for unraveling protein function in diverse cellular environments.^34,35^

Our study presents a deep learning approach that integrates PLMs with multiwindow scanning technology for protein sequence analysis, which we call NA_mCNN for classifying sodium transporters. To classify sodium transporters from membrane proteins more accurately, this study examined pretrained protein language models such as Tape, ProtTrans, ESM-1b, and ESM-2.^29,36−38^ This model has been demonstrated to be highly accurate and reliable for identifying sodium transporters from membrane proteins. This has significant implications for drug targeting in metabolic and cardiovascular diseases, such as diabetes, heart failure, and hypertension. The focus of our research on sodium transporters is the functional relevance of these transporters to advance our understanding of their physiological and pathological implications.

## 2 Materials and Methods

**Figure 2:**
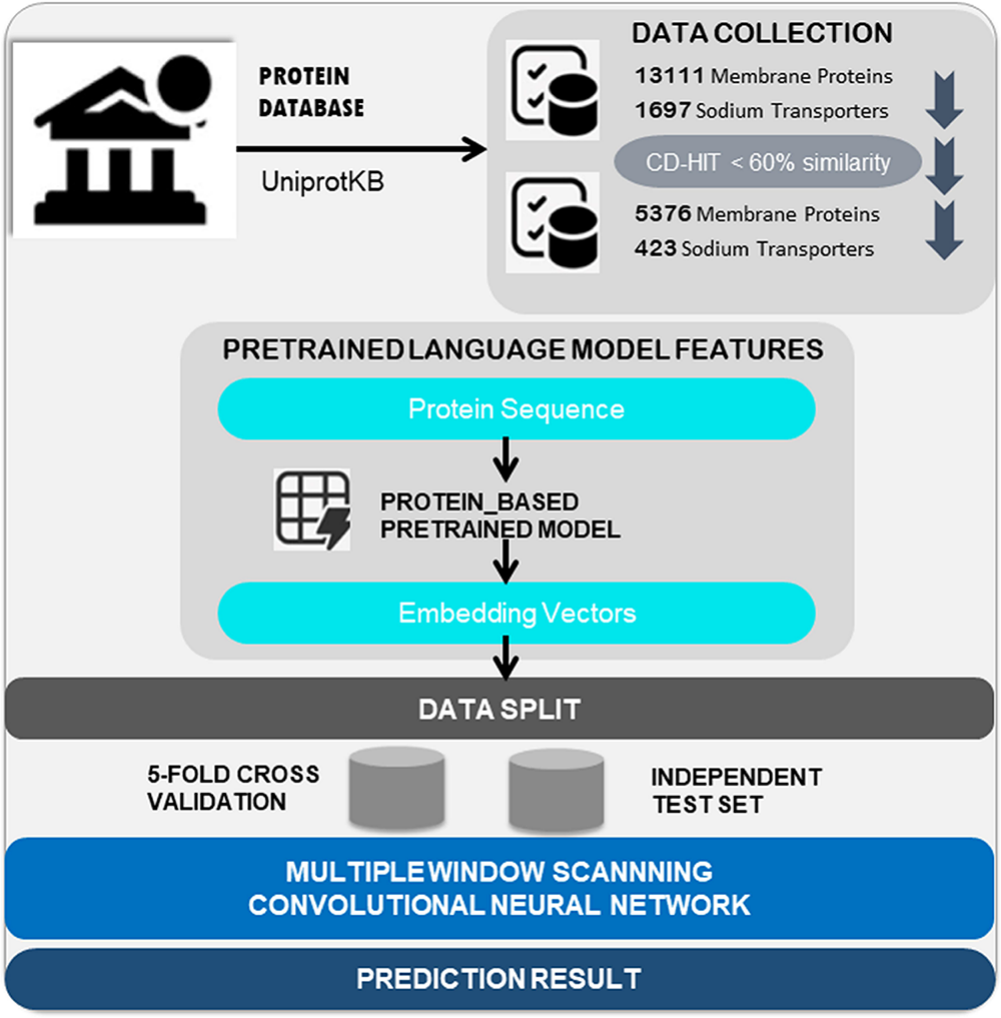
NA_mCNN Model: An architecture work flow depicting data gathering and preprocessing, feature extraction with PLMs, and the implementation of advanced deep learning network.

Figure 2 depicts how NA_mCNN combines deep learning methodologies with state-of-the-art language models to optimize functional classification of sodium transporters. The details about our data acquisition, feature extraction, and advanced deep learning networks are as follows.

### 2.1 Data Collection

In the data set, we collected sodium transporters and membrane proteins data from Uniprot.^39^ Specifically, sodium transporters located in plasma membranes were identified using the query, and a detailed description of the data collection and preprocessing methods can be found in the Supporting Information (Supporting Information S1–S5). This data set contains 1697 sequences belonging to the sodium transporters class and 13,111 sequences belonging to the membrane proteins class. To maintain data integrity, we applied stringent criteria to improve data quality, excluding sequences with fewer than 50 residues. By eliminating redundancy and ensuring that the data set contains a diverse but representative set of sequences, the CD-HIT^40^ step is crucial for subsequent analysis. As a result of applying CD-HIT with a threshold of 60% similarity, the number of distinct sequences of the sodium transporters class and membrane proteins class is reduced to 423 and 5376, respectively. To ensure a representative distribution of classes, 80% of the sequences in both data sets were assigned to the training set and the remainder to the test set. A total of 338 sequences were assigned to the sodium transporters class, while 4300 sequences were assigned to the membrane proteins class. In a test set, 85 sodium transporters and 1076 membrane proteins were assigned, as shown in Table 1. Our study used a sequence similarity threshold of 60% to ensure sufficient diversity in the data set while capturing functional similarities effectively. A classification threshold of 60% provided sufficient samples for training without sacrificing significant functional characteristics. In accordance with Tian and Skolnick,^41^ 60% sequence identity can be used to predict function, Addou et al.^42^ recommended a 60% threshold for functional annotations for maintaining functional consistency across homologous sequences. In addition, Ahmed et al.^43^ demonstrated 72.7% balanced distribution with a 60% similarity threshold while maintaining 2% sequence removal. However, 60% of similarity studies remain robust despite achieving significant functional insights.

**Table 1:** Data Collection and Pre-Processing of Sodium Transporters and Membrane Proteins

	original	similarity < 60%	train	test
Class Name				
sodium transporters	1697	423	338	85
membrane proteins	13,111	5376	4300	1076
total	**14,808**	**5799**	**4638**	**1161**
Additional Test Set				
sodium transporters	9	9		9
membrane	228	95		95
total	**237**	**95**		**104**

We added newly discovered sodium transporter sequences from the UniProt database (August 2024) 9 sodium transporter sequences were identified in the UniProt database and published or annotated, to test our model’s generalization ability. We selected enough membrane samples randomly so that no overlap occurred between the training and validation data sets, and we used recently discovered membrane protein sequences obtained from the UniProt database after the original data set had been collected. Based on Table 1, the final additional test set included 9 positive and 95 negative samples, each evaluated independently to ensure that new data would not be found in the training and validation sets.

### 2.2 Feature Extraction

A variety of techniques have been used to extract informative features from protein sequences. One-hot encoding is one of the most common methods for encoding protein sequences, but it may not capture sodium transporters’ complex sequence patterns. Multiple Sequence Alignment (MSA) is another method of identifying conserved positions in a protein based on the evolutionary relationship between it and its homologues.^44^ However, to construct MSAs for a large database of protein families, it is necessary to put a considerable amount of computational power into the process.

Currently, several pretrained language models that combine natural language processing with protein sequence analysis have been developed to provide computer-aided natural language analysis.^45−47^ Neural networks have been demonstrated to provide insight into complex patterns within large text sets in several models, including Transformers, BERT, GPT-3, and ReBERTa.^26−28,48^ Additionally, the evolution of these pretrained protein language models (PLMs) has also been reflected in bioinformatics, where researchers use PLMs for a wide variety of tasks in their research. To train these models, extensive sequence data is used to extract rich embeddings that reflect nuanced interactions between amino acids within protein sequences.

In our study, we used language model embeddings to understand complex molecular structures by extracting features from protein sequences with cutting-edge techniques. To gain insight into intricate sequence patterns and interrelationships, we use five different language model embeddings, including Tape, ESM-1b, ESM-2, ProtTrans, and ProstT5.^29,36−38,49^ Our framework for unraveling protein sequence complexity aims to advance research and practical application at the same time by rigorously evaluating these language model embeddings.

#### 2.2.1 ProtTrans Model for Protein Languages

The integration of language models into protein sequences has transformed bioinformatics, providing researchers with a powerful tool for decoding the complex language encoded within proteins. As part of Elnaggar et al.’s groundbreaking research,^29^ ProtTrans leverages self-supervised learning, a machine learning paradigm where the model learns from the inherent structure of data without explicit supervision. A large data set of protein sequences encompassing diverse proteins from various organisms is required for training ProtTrans, which is often obtained from UniProt databases. To capture different aspects of protein sequence information, including local and global context, ProtTrans employs a variety of self-supervised learning tasks during training. Once trained, ProtTrans can generate embeddings for newly discovered protein sequences. This study utilizes the ProtTrans model “Rostlab/prot_t5_xl_half_uniref50-enc,” consisting of 24 layers, approximately 1.2 billion parameters, and 1024 embedding dimensions.

As a first step in extracting features from our data set, we encoded each protein sequence into a ProtTrans embedding that contained crucial contextual and sequence information. Because sodium has a context-specific function, this language model captures subtle differences in protein function. Each embedded feature has a constant dimension of 1024 throughout the data set, providing uniform and structured representations.

#### 2.2.2 Evolutionary Scale Modeling (ESM-1b)

ESM-1b, a variant of evolutionary scale modeling (ESM),^37^ attempts to capture intricate patterns and relationships within complex biological structures. The use of a large and meticulously crafted protein sequence model allows us to uncover previously unknown features and relationships. The ESM-1b embeddings provide contextual information and allow patterns to be encoded in a higher dimension, which makes them crucial to feature extraction. The model consists of 33 layers, 650 million parameters, and 1280 embedded dimensions. Therefore, ESM-1b can contribute to a more comprehensive and in-depth understanding of these highly relevant biological molecules.

#### 2.2.3 Evolutionary Scale Modeling (ESM-2)

ESM-2 embeddings are generated by the ESM-2 model, a variant of Evolutionary Scale Modeling (ESM) used for learning protein sequence representations.^38^ In bioinformatics and computational biology, these embeddings are employed in various downstream tasks that capture protein sequence essential features. To extract different features from another protein language, we continuously applied ESM-2, which is a deep-learning model for modeling protein languages. By using ESM-2, we can explore protein sequences at greater depth since it consists of 33 layers with 650 million parameters and 1280 dimensions. Our analysis is based on ESM-2 embeddings, which provide context and encoding for more complex patterns. By incorporating ESM-2 into our research, we can improve our understanding of these critical biological molecules while also increasing the complexity and depth of our molecular analysis.

#### 2.2.4 Tape Embeddings

Transformer Architecture for Protein Embedding (TAPE),^36^ a pioneering approach to protein sequence analysis, is renowned for its robustness and versatility. TAPE encodes amino acids using a 768-dimensional embedding scheme that captures protein sequence essence in rich and expressive representations. To process natural language, TAPE uses transformer-based models, which are well-known for their effectiveness. In addition to encapsulating essential sequence information, it facilitates efficient processing and analysis. The TAPE system extracts nuanced features of protein structure and function by capturing long-range dependencies within protein sequences. In addition, TAPE’s representations are robust and comprehensive because it has been trained on large protein sequence data sets. The main advantage of TAPE is its ability to learn protein sequences without labeling them using advanced self-supervised learning techniques, such as masked language modeling and next-sentence prediction. A major benefit of this method is that it provides insight into the way proteins function, evolve, and interact, which is useful for drug discovery, bioengineering, and precision medicine.

#### 2.2.5 ProstT5 Embeddings

With ProstT5,^49^ protein structure and function can be predicted more accurately using information from 3D structural models (3Di) and amino acid sequences. ProstT5 extends ProtT5′s methodology by mapping AA sequences to tokens that represent structural contexts, such as helices, strands, and loops. Because this model captures structural and sequence context, it can be used for downstream prediction tasks such as identifying ligand binding sites and secondary structures. Using 34 million samples obtained from both AlphaFold2 predictions and experimentally verified protein structures, they trained ProstT5 on a T5 transformer model to predict masked sequences. During training, ProstT5 optimized “bilingual” embeddings to learn both AA and 3Di representations, thus providing a more accurate understanding of structural relationships. ProstT5′s embedded size is 1024, ensuring compatibility with other protein language models and simplifying integration.

### 2.3 Model Architecture

**Figure 3:**
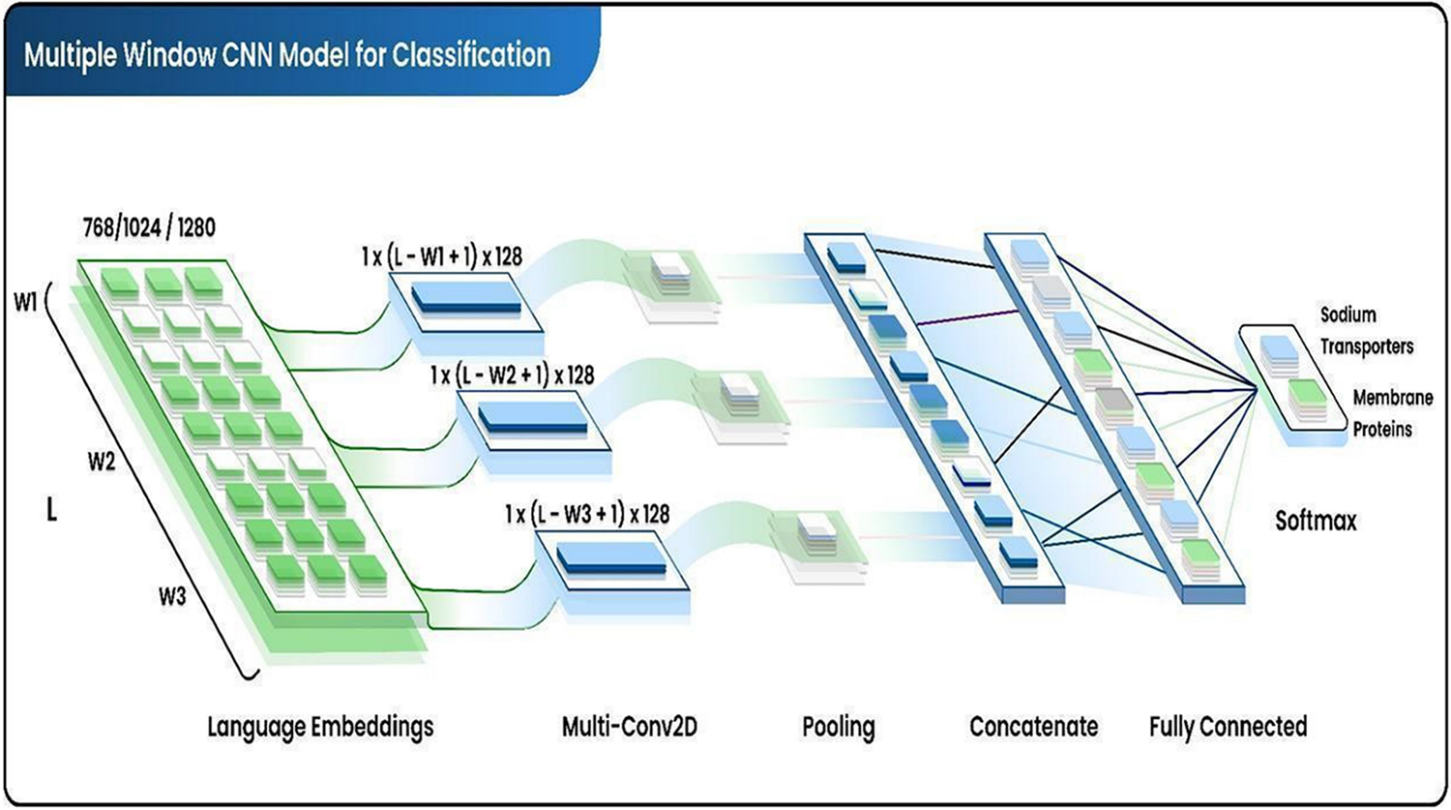
Multiple-window scanning deep learning networks. Multiple window convolutions and filters are applied to embedded language, which is then pooled and finally concatenated and passed through fully connected layers.

The multiwindow convolution filter was used to scan the contents of the 3D matrix (1 × *L* × 1024), where *L* represents the sequence length and 1024 denotes the embedded dimensions of each amino acid. The main feature of this model is its multiwindow convolutional scanning mechanism that utilizes convolutional layers with varying window sizes (e.g., 16 × 1 × 1024 kernels) to capture both local and global sequence patterns. During each convolutional layer, the 3D input is transformed into a 2D matrix (*L* × 1), and the most dominant features are extracted using max pooling. Feature integration combines outputs from different window convolutions to create a comprehensive feature vector that represents cross-scale patterns. Protein language models provide a foundation for this architecture, which extracts salient features efficiently across multiple scales and provides a detailed description of sequences. The architecture of our convolutional neural network is shown in Figure 3. During the training process, this model was tested on a GeForce RTX 3090 GPU.

Our training strategy avoided overfitting and ensured high performance for NA_mCNN. We used ReLU activation to train convolutional layers of varying window sizes, max-pooling, dense layers, and a dropout rate of 0.7 to prevent overfitting. A final dense layer was optimized using L2 regularization (1 × 10^–3^). Dynamic learning rate adjustments were made with 0.001 in the initial learning rate. In training, the model was stopped early based on validation loss to ensure robustness.

## 3 Performance Evaluation

Our model is evaluated using several established metrics, including threshold-dependent metrics such as sensitivity, specificity, accuracy, and Matthew’s correlation coefficient (MCC). The sensitivity of a model is determined by the number of positive samples that are correctly classified. The specificity of a model determines whether it is capable of accurately classifying negative cases. Classification accuracy is defined as the proportion of correctly predicted samples out of all instances evaluated. By using the MCC, we can evaluate the accuracy of data sets in a more nuanced manner, considering both positives and negatives. Furthermore, the F1-Score indicates that our model can identify true positives while minimizing false positives.

One of the most effective ways to assess classification performance across a wide range of thresholds is to use threshold-independent metrics such as the area under the receiver operating characteristic curve (AUC-ROC). To determine whether a binary classification model can effectively discriminate between classes, AUC-ROC curves are used.

Additionally, all the parameters associated with eqs 1–5 were thoroughly calculated to allow for a rigorous evaluation of these equations.
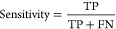
1
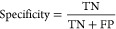
2
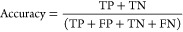
3
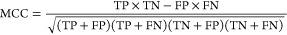
4
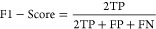
5TP stands for true positives, TN stands for true negatives, FP reflects false positives, and FN indicates false negatives.

## 4 Results

To evaluate the effectiveness of our computational model in classifying sodium transporters from membrane proteins, we conducted a 5-fold cross-validation method and independent tests, which proved its ability to predict sodium transporters from membrane proteins. We further tested our model with other machine learning classifiers to assess its predictive accuracy. Accordingly, it is reasonable to conclude that our approach has the potential to be both robust and effective for the classification of biologically relevant proteins.

### 4.1 Analysis of Single Window Sizes

The model’s performance metrics across a wide range of window sizes are valuable. The ability of a model to correctly detect and predict outcomes is measured by metrics such as sensitivity, specificity, accuracy, and the Matthews correlation coefficient (MCC). Table 2 shows that window size (2) provides the best balance across all metrics, making it the optimal first choice. It is characterized by a high sensitivity of 0.9765 and a specificity of 0.9963, resulting in an overall accuracy of 0.9948. In addition, with a Matthews correlation coefficient (MCC) of 0.9624 and an area under the curve (AUC) of 0.9990, it demonstrates a strong ability to handle imbalanced data sets. Second, window size (16) has the highest sensitivity of 0.9882, which is crucial for detecting the minority class (sodium transporters), but its MCC of 0.9458 and AUC of 0.9965 are slightly lower than those of window size (2). In light of this result analysis, window size (2) should be prioritized when sodium transporter detection is of high importance, whereas window size 16 could be a suitable alternative when balanced classification is more important.

**Table 2:** Evaluation of Sodium Transporters Classification Using Different Single Window Sizes

window size	sensitivity	specificity	accuracy	MCC	AUC
**2**	**0.9765**	**0.9963**	**0.9948**	**0.9624**	**0.9990**
4	0.9765	0.9954	0.9940	0.9564	0.9983
8	0.9765	0.9926	0.9914	0.9392	0.9985
16	0.9882	0.9926	0.9922	0.9458	0.9965
32	0.9882	0.9898	0.9897	0.9294	0.9979
64	0.9647	0.9963	0.9940	0.9558	0.9981

### 4.2 Analysis of Multiple Window Combinations

According to the performance metrics listed in Table 3, we compared different window combinations for classification based on different parameter settings for the given metric result. Based on the analysis of different window combinations, the combination (4,8,16) has the best overall performance, making it the most appropriate choice for this study. As a result of this combination’s sensitivity of 0.9765 and specificity of 0.9944, both minority sodium transporters and majority membrane proteins are detected effectively. As illustrated by the high accuracy of 0.9931 and the MCC of 0.9506, there is a strong correlation between predicted and actual classifications, while the excellent model discrimination is demonstrated by the AUC of 0.9990. In contrast, the other combination (4,16) shows the highest sensitivity of 0.9882, which makes it ideal in scenarios where sodium transporters are of primary importance. Despite a slightly lower MCC of 0.9458, the AUC of 0.9990 assures reliable model performance. Other combinations, such as (2,8,16) and (4,8,16,32), showed lower specificity and MCC, suggesting that additional windows do not improve performance. Therefore, the combination of (4,8,16) is the most balanced and robust option for the sodium transporters classification.

**Table 3:** Evaluation of Sodium Transporters Classification Using Different Multiple Window Combinations

window size	sensitivity	specificity	accuracy	MCC	AUC
(4 16)	0.9882	0.9926	0.9922	0.9458	0.9990
(2 8 16)	0.9882	0.9833	0.9836	0.8940	0.9988
**(4 8 16)**	**0.9765**	**0.9944**	**0.9931**	**0.9506**	**0.9990**
(4 8 16 32)	0.9882	0.9898	0.9897	0.9294	0.9965
(4 8 16 32 64)	0.9882	0.9870	0.9871	0.9138	0.9964

### 4.3 Analysis of Varying Filter Sizes

Table 4 compares the performance of a model with different filter sizes, 64, 128, 256, and 512, across a variety of key metrics. The evaluation of model performance of 64 filters offers the most effective overall results for handling the imbalanced data set, providing the strongest correlation between predicted and actual classifications. In addition to providing the highest overall results, this filter size also demonstrated an excellent accuracy of 0.9974. It also demonstrated the highest specificity of 0.9991 and the best MCC of 0.9746. Although other configurations, such as 256 filters, do perform well, with an MCC of 0.9685 and a slightly lower specificity of 0.9972, 64 filters continue to perform better in terms of overall balance. 512 filters exhibit the highest sensitivity of 0.9882 but the lowest MCC of 0.9294, suggesting a trade-off between overall performance and sensitivity. Therefore, 64 filters are the most appropriate choice for this study, as they effectively balance sensitivity, specificity, and MCC.

**Table 4:** Evaluation of Sodium Transporters Classification Using Different Filter Sizes

filter size	sensitivity	specificity	accuracy	MCC	AUC
**64**	**0.9765**	**0.9991**	**0.9974**	**0.9746**	**0.9975**
128	0.9765	0.9944	0.9931	0.9506	0.9990
256	0.9765	0.9972	0.9957	0.9685	0.9988
512	0.9882	0.9898	0.9897	0.9294	0.9989

### 4.4 Analysis of 5-Fold Cross Validation with Different Pre-Trained Language Model Embedding

**Figure 4:**
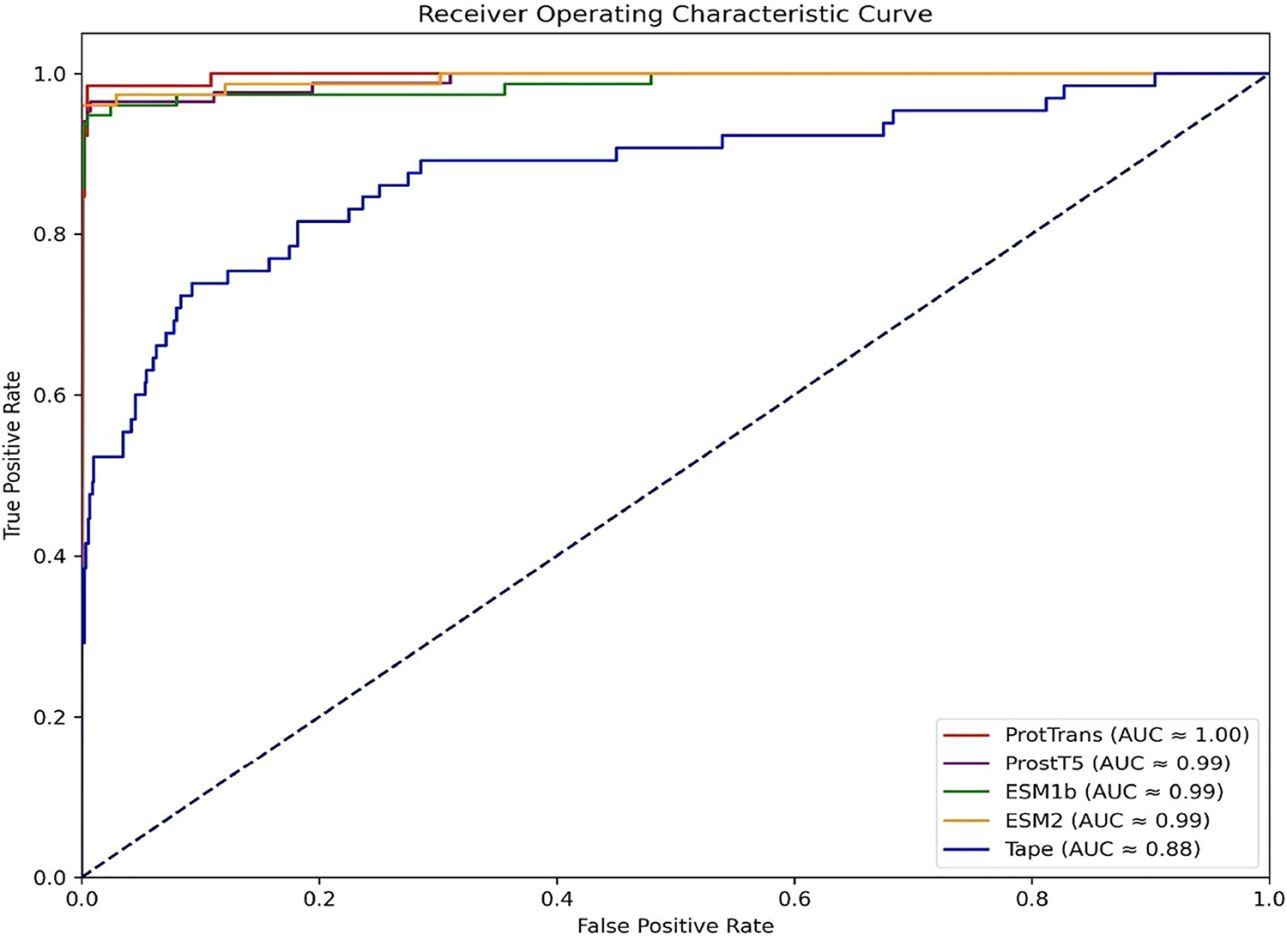
AUC-ROC curve comparison between different pretrained proteins language models feature set for sodium transporters classification.

The 5-fold cross-validation results of different pretrained language model embeddings in Table 5 indicate that ProtTrans and ESM2 have the highest overall performance. ProtTrans has a sensitivity of 0.9829, and ESM2 follows at 0.9673. While ESM2 has a marginally higher specificity of 0.9924 and MCC of 0.9309, the differences are not significant. Additionally, ProtTrans has a smaller embedding vector size of 1024 compared to ESM2 of 1280, making it more computationally efficient. ProtTrans offers near-equivalent predictive power to ESM2 due to its efficiency advantage, coupled with its strong AUC of 0.9939, making it an attractive choice. In contrast, Tape exhibits significantly lower sensitivity of 0.7408 and MCC of 0.4289 due to its lighter embedding, indicative of its limited ability to handle unbalanced data sets. As a result of its AUC of 0.8785, it lacks the discriminatory power necessary for accurate classification as compared to ProtTrans and ESM2. Besides ProtTrans, ESM1b, and ESM2, we also used ProstT5, a structure-aware protein language model that encodes 3D structural relationships between amino acids. ProstT5 achieved comparable specificity of 0.9886 and accuracy of 0.9868 but with a lower sensitivity of 0.9647 and MCC of 0.9125. It also maintained excellent specificity and F1-Score. Despite these small differences, ProstT5 remains competitive, providing valuable structural insights for sodium transporter classification. Considering the balance between high performance and computational efficiency, ProtTrans is the best embedding for this study. The ROC curves for these comparisons are shown in Figure 4.

**Table 5:** Evaluation of Sodium Transporters Classification Using 5-Fold Cross Validation with Different Pre-Trained Language Model Embedding

feature sets	sensitivity	specificity	accuracy	MCC	F1-Score	AUC
Tape	0.7408	0.8748	0.8653	0.4289	0.4478	0.8785
ESM1b	0.9385	0.9780	0.9752	0.8333	0.8414	0.9854
ESM2	0.9673	0.9924	0.9905	0.9309	0.9349	0.9932
ProstT5	0.9647	0.9886	0.9868	0.9125	0.9169	0.9912
**ProtTrans**	**0.9829**	**0.9889**	**0.9884**	**0.9209**	**0.9239**	**0.9939**

### 4.5 Analysis of an Independent Test Set with Different Pre-Trained Language Model Embedding

In the independent test for sodium transporter classification using different pretrained language model features, as illustrated in Table 6, ProtTrans and ESM1b were found to be the most accurate embeddings. Based on ProtTrans’s high specificity and high sensitivity, a strong MCC of 0.9746 and an AUC of 0.9975 indicate its remarkable ability to identify both positive and negative samples correctly. While ESM1b provides excellent discriminatory power, ProtTrans slightly outperforms it in terms of specificity and AUC, with a sensitivity of 0.9765, a specificity of 0.9944, a MCC of 0.9506, and an AUC of 0.9948. However, ESM2 exhibits a slightly lower specificity of 0.9842 and MCC of 0.8988 than ProtTrans and ESM1b, which may introduce more false positives. Tape displays the lowest performance with a sensitivity of 0.7647, specificity of 0.8253, MCC of 0.3723, and AUC of 0.8743, which indicates moderate discriminatory capability but is not comparable. With its 3D structure aware feature, ProstT5 achieved similar specificity and accuracy of 0.9926 and 0.9905, however with a lower sensitivity of 0.9647, and also maintained an impressive MMC of 0.9325 and F1 score of 0.9371 respectively. Although ProstT5 has some differences, it remains competitive, providing useful structural insights. This study indicates that ProtTrans shows the most effective combination of sensitivity and specificity while also providing superior computational efficiency due to the small embedding vector size.

**Table 6:** Evaluation of Sodium Transporters Classification Using Independent Test Set with Different Pre-Trained Language Model Embedding

feature sets	sensitivity	specificity	accuracy	MCC	F1-score	AUC
Tape	0.7647	0.8253	8208	0.3723	0.3846	0.8743
ESM1b	0.9765	0.9944	0.9931	0.9506	0.9540	0.9948
ESM2	0.9882	0.9842	0.9845	0.8988	0.9032	0.9951
ProstT5	0.9647	0.9926	0.9905	0.9325	0.9371	0.9926
**ProtTrans**	**0.9765**	**0.9991**	**0.9974**	**0.9746**	**0.9333**	**0.9975**

### 4.6 Analysis of Sodium Transporters Classification with Different Machine Learning Models

**Figure 5:**
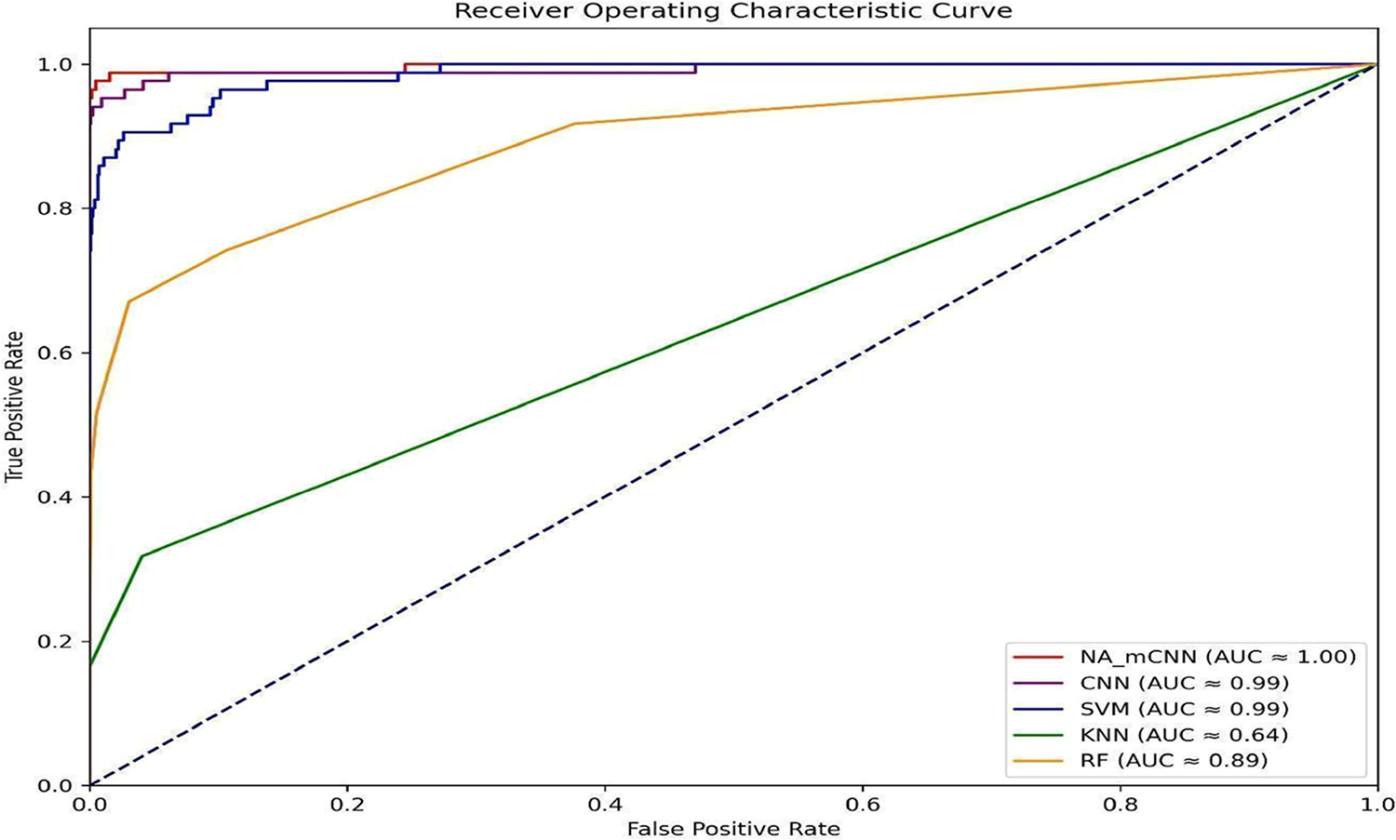
AUC-ROC curve comparison between NA_mCNN and different machine learning models.

The NA_mCNN of multiwindow scanning CNN models outperform all other machine learning models in predicting sodium transporters within an imbalanced data set, as shown in Table 7. The KNN and RF have high specificities of 1.0000 and 0.9991, but their sensitivity is low, especially the KNN of 0.1647. The SVM is able to effectively handle minority classes by achieving a sensitivity of 0.7882 and a specificity of 0.9981. Compared to the traditional machine learning models, CNN performed best in predicting both classes correctly, achieving an accuracy of 98.79%, a Matthew’s correlation coefficient of 0.9146, and an F1 score of 0.9205. We strategically selected the number of filters and hidden layers for the CNN model based on our multiwindow scanning analysis. NA_mCNN, on the other hand, performs exceptionally well across all metrics, in which it has near-perfect sensitivity of 0.9965, specificity of 0.9991, accuracy of 0.9974, and an MCC of 0.9944, F1-Score of 0.9333, and AUC of 0.9975. NA_mCNN is the most effective model for detecting the underrepresented positive class as well as other parameters in this imbalanced sodium transporter classification task. Figure 5 shows the AUC-ROC curves for these comparisons.

**Table 7:** Evaluation of Sodium Transporters Classification Using Different Machine Learning Models

classifier	sensitivity	specificity	accuracy	MCC	F1_score	AUC
KNN	0.1647	1.0000	0.9388	0.3931	0.2828	0.6417
RF	0.3412	0.9991	0.9509	0.5586	5043	0.8928
SVM	0.7882	0.9981	0.9828	0.8664	0.8701	0.9859
CNN	0.9529	0.9907	0.9879	0.9146	0.9205	0.9928
**NA_mCNN**	**0.9765**	**0.9991**	**0.9974**	**0.9746**	**0.9333**	**0.9975**

### 4.7 Validation of Novel Sodium Transporters

To test the generalizability of our model, we used an additional test set of sodium transporter sequences (*n* = 9) and membrane protein samples. The model has also demonstrated robustness and generalizability on the new test set, which includes additional sodium transporter sequences. The model maintained a high sensitivity of 1.0000, specificity of 0.9789, accuracy of 0.9808, MCC of 0.8950, F1-Score of 0.9000, and AUC of 0.9813 despite only having 9 additional positive samples. This demonstrates its ability to generalize effectively to previously unseen data. Based on these findings, it is demonstrated that the model can identify newly discovered sodium transporters.

### 4.8 Model Interpretation of the NA_mCNN

**Figure 6:**
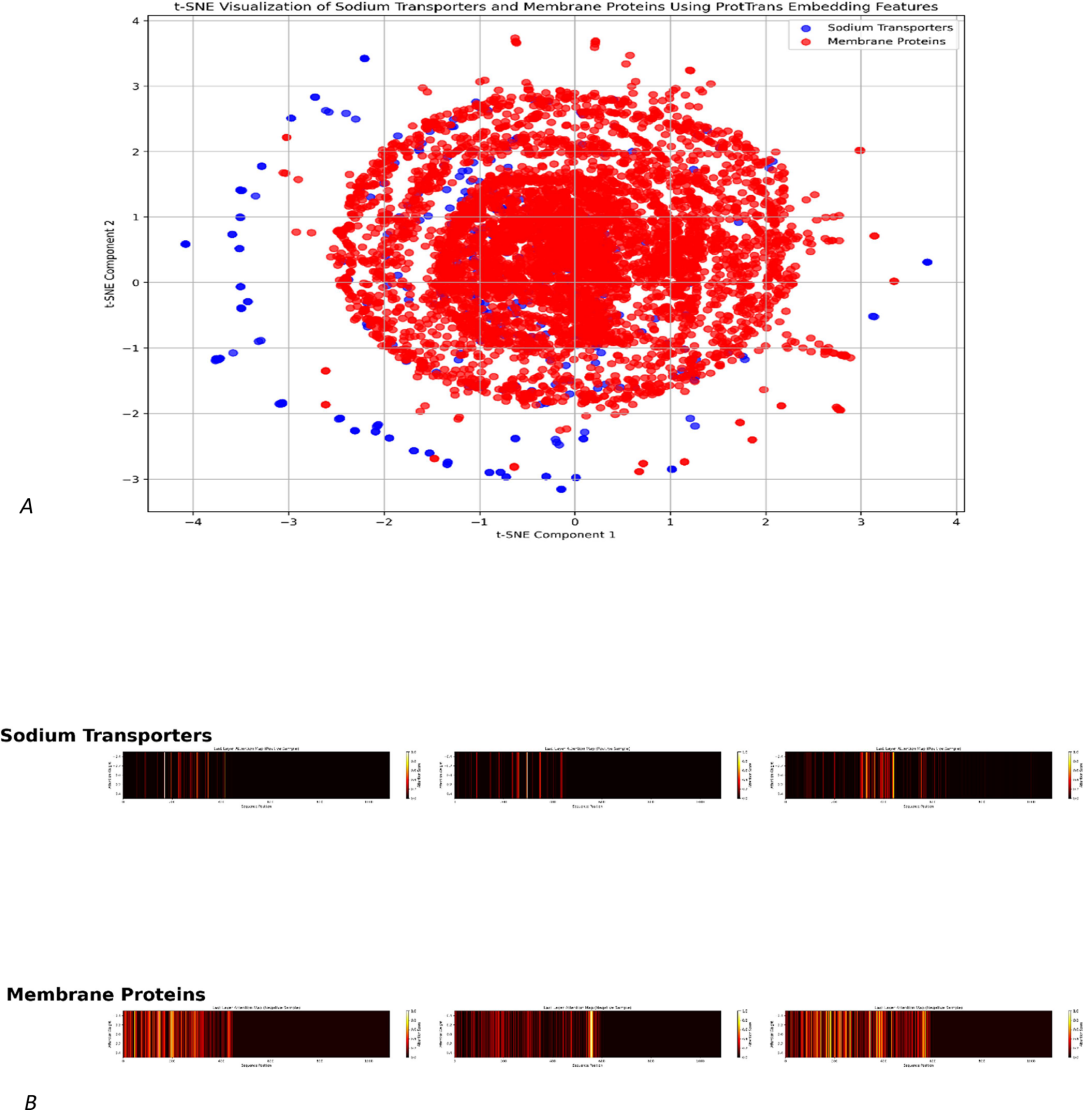
Presentation of the interpretability of the results. (A) is the feature visualization of the ProtTrans embedding by the t-SNE. (B) is the visualization of Grad-CAM heatmap highlighting the model’s attention on sodium transporters and membrane proteins.

The Na_mCNN architecture was evaluated by extracting intermediate layers from each stage of the model and mapping two-dimensional features to the intermediate layers. As illustrated in Figure 6A of the ProtTrans embedding, the t-SNE plot indicates a significant overlap between sodium transporters (blue) and membrane proteins (red), indicating that their properties do not differ significantly in the embedding space. Although membrane proteins are grouped closely, sodium transporters are more randomly distributed and intermittently scattered, indicating greater variability. Blue dots indicate outliers with specific sodium transporter properties may be difficult to identify solely based on ProtTrans embeddings, however the model captures sodium transporter characteristics accurately.

In addition, Gradient-weighted Class Activation Mapping (Grad-CAM) was used to interpret the features learned by the model’s convolutional layers, highlighting the most significant regions within the classification sequences. Convolutional layers likely capture conserved functional domains in sodium transporters, which are biologically significant. As shown in Figure 6B, sodium transporters have narrow activation regions, particularly around specific sequence positions, indicating that the model relies on important motifs or functional residues. By contrast, membrane proteins have broader and more dispersed activation patterns, which reflect their diverse structural and functional elements, like transmembrane helices and loops. The model appears to be effective in distinguishing between the two classes of proteins, taking advantage of the localized and precise features of sodium transporters, and utilizing the more complex and heterogeneous sequence information on membrane proteins. Based on the comparative analysis, it appears that the model can identify features that are specific to classes of proteins as well as adaptable to a wide variety of sequences.

## 5 Discussion

The NA_mCNN method uses cutting-edge pretrained protein language models and deep learning convolutional neural networks to classify sodium transporters in membrane proteins. Drug development focuses on sodium transporters, such as sodium-glucose cotransporters (SGLTs) and sodium–potassium–chloride cotransporters (NKCCs), since these proteins are implicated in several diseases, including diabetes, hypertension, and neurological disorders. By identifying and characterizing sodium transporters, we can improve drug discovery efforts, gain a deeper understanding of their roles, and learn how sodium-dependent SLC proteins contribute to diabetes, cardiovascular disease, and neurological disorders. Computational classification of these transporters contributes to a deeper understanding of their physiological functions and therapeutic potential.

Our study examined and optimized multiple parameters, including window sizes, window combinations, filter sizes, and different embeddings of pretrained protein-language models. ProTrans emerged as the most impressive feature set. We achieved high accuracy, sensitivity, and AUC of 0.9884, 0.9829, and 0.9939, respectively, for classifying sodium transporters from membrane proteins as 5-fold cross validation. Our models were able to predict the same protein class as an independent test set with accuracy, sensitivity, and an AUC of 0.9974, 0.9765, and 0.9975, respectively, showing robustness and effectiveness. Through multiple window scanning and language models, our model has been rigorously tested for generalization. The model performed consistently across cross-validation and independent tests, demonstrating its ability to classify unknown data accurately.

Our study, which combines ProtTrans features with a multiwindow scanning CNN model, produces excellent validation metrics (MCC: 0.8950, AUC: 0.9813) in novel proteins for sodium transporter classification, with direct biological implications. The model identifies conserved structural elements required for sodium transport function, which sheds light on how conformational changes contribute to pathological conditions. For example, the model’s high accuracy (0.9808) in recognizing structural signatures contributes to a better understanding of Na+/K+-ATPase changes in neurological disorders and transporter variations that affect glucose transport in diabetes. Beyond classification, balanced prediction reliability (F1-Score: 0.9000) makes it easier to identify potential drug-binding sites and novel transporters in genomic data, bridging the gap between computational prediction and biological function and speeding up therapeutic development.

A limitation of the study is the use of a 40% similarity threshold, which resulted in a significant reduction in positive sequences and a reduction in performance metrics. There is a trade-off between data set size and sequence similarity, which limits the ability of the model to detect meaningful patterns. The future research should examine alternative methods for expanding the data set while maintaining low similarity thresholds, such as incorporating additional data from multiple sources or developing models that can learn effectively from smaller, more distinct data sets.

This analysis highlights ProtTrans’ superior performance, particularly in sensitivity and MCC, compared to other feature sets in accurately classifying sodium transporters. Our research extends beyond bioinformatics to a wide range of disciplines, including cell biology and membrane protein functions, by examining the roles and interactions of protein classes such as sodium transporters. By utilizing our NA_mCNN model, scientists can gain a deeper understanding of complex cellular processes, which will help understand health and disease. Our research contributes to the ever-expanding body of scientific knowledge by advancing our understanding of cellular mechanisms, which will pave the way for future discoveries.

## 6 Conclusions

The NA_mCNN Model, consisting of pretrained protein language models, was used in conjunction with a multiwindow scanning CNN model to classify sodium transporters and predict SLC proteins belonging to the same functional class. Our objective with these advanced computational techniques was to gain a deeper understanding of sodium transporters’ role in health and disease management, particularly their classifications and prediction patterns.

The NA_mCNN Model, consisting of pretrained protein language models, was used in conjunction with a multiwindow scanning CNN model to classify sodium transporters. Our objective was to gain a deeper understanding of sodium transporters’ structures and functions through the classification of previously unannotated or unexplored protein sequences. In this manner, sodium transporters can be annotated and characterized, providing the foundation for future research and drug discovery efforts.

ProtTrans embedding proves to be effective and suitable for this study based on the results from this experiment. The performance of ProtTrans was strong both in 5-fold cross-validation and in an independent test set. In addition to ESM1b, ESM2, and prostT5, all three embeddings performed extremely well, with results that are close to ProtTrans in many metrics. However, considering the balance between high performance and computational constraints, ProtTrans was chosen for its optimal blend of accuracy, efficiency, and practicality in handling protein sequence classification tasks.

This study contributes to a better understanding of sodium transporters, a critical component of human health and disease, as sodium transporter dysfunction and disease progression not only reveal pathological mechanisms but also provide new avenues for pharmacological manipulation, potentially revolutionizing hypertension, diabetes, and neurological conditions. NA_mCNN can be accurately identified by combining pretrained protein language models with multiscanning window convolutional neural networks with comprehensive classification accuracy. Our findings highlight potential therapeutic targets as well as contributing to precision medicine and facilitating faster drug discovery processes.

## Data Availability

Our code and data set can be found on GitHub: https://github.com/Malik-glt/DeepSodiumTransporters

## Supporting Information Available

UniProt query for sodium transporters; membrane protein data set collection details; summary of data set statistics; sequence length selection rationale and methodology; and CD-HIT clustering strategy and its impact on data set integrity (PDF)
