# Supplementary material for: NA_mCNN: Classification of Sodium Transporters in Membrane Proteins by Integrating Multi-Window Deep Learning and ProtTrans for Their Therapeutic Potential
Source: J Proteome Res. 2025 Apr 7;24(5):2324–35. doi: 10.1021/acs.jproteome.4c00884 (PMC12053934; doi:10.1021/acs.jproteome.4c00884)
Supplement: Supplementary file 1 — pr4c00884_si_001.pdf [file pr4c00884_si_001.pdf]

NA_mCNN: Classification of Sodium Transporters in Membrane Proteins by Integrating Multi-Window Deep Learning and ProtTrans for Their Therapeutic Potential

Muhammad Shahid Malik,[1,3] Van The Le,[1] Yu-Yen Ou,[1,2*]

[1] Department of Computer Science and Engineering, Yuan Ze University, Chung-Li- 32003, Taiwan
[2] Graduate Program in Biomedical Informatics, Yuan Ze University, Chung-Li- 32003, Taiwan
[3] Department of Computer Sciences, Karakoram International University, Gilgit-Baltistan - 15100, Pakistan

*Corresponding author: yien@saturn.yzu.edu.tw

Table of Contents

- **Supplementary Material S1:**
  UniProt query for sodium transporters
- **Supplementary Material S2:**
  Membrane protein dataset collection details
- **Supplementary Material S3:**
  Table S1 – Summary of dataset statistics
- **Supplementary Material S4:**
  Sequence length selection rationale and methodology
- **Supplementary Material S5:**
  CD-HIT clustering strategy and its impact on dataset integrity

Supplementary Material S1:

UniProt Query for Sodium Transporters:

In this study, we collected data on sodium transporters and membrane proteins from UniProt using the following query:
(cc_scl_term:SL-0162) AND (keyword:KW-0739) AND (reviewed:true) AND (precursor:false) AND (fragment:false)

Supplementary Material S2:

Membrane Protein Dataset Collection:

The membrane proteins we collected dataset from the paper *. All sequences included in the dataset were carefully filtered and only reviewed entries from the data set that are not precursor or fragment sequences. A detailed summary of the data collection results, including the number of proteins, is provided in the accompanying table.

Supplementary Material S3:

Summary of Dataset Statistics: Table S1.

**Class**	**Sequences**	**60% Similarity**	**80% Train**	**20% Test**
Sodium Transporters	1697	423	338	85
Membrane*	13111	5376	4300	1076
**Total**	**14808**	**5799**	**4638**	**1161**

***Shahid Malik, M.; Ou, Y.-Y. Integrating Pre-Trained protein language model and multiple window scanning deep learning networks for accurate identification of secondary active transporters in membrane proteins. *Methods* **2023**, *220*, 11– 20, DOI: 10.1016/j.ymeth.2023.10.008

Supplementary Material S4:

Sequence Length Selection:

For this study, we selected a **sequence length cutoff of 1100** amino acids. This decision was based on an analysis of the dataset, **where we found that 90% of the sequences were under 1100 amino acids in length**. By setting the sequence length at 1100, we ensure that the majority of the data is captured without introducing excessive padding or truncation, which could negatively affect model performance. This length was chosen to maintain the integrity of the protein sequences while minimizing unnecessary computational complexity. It also reflects the typical length range for sodium transporters and membrane proteins, ensuring biologically relevant features are retained for accurate classification.

Supplementary Material S5:

CD-HIT Clustering to reduce the Similarity

We chose a 60% similarity threshold for CD-HIT clustering in order to reduce redundancy in the dataset while maintaining sufficient sequence diversity. This threshold strikes a balance between

retaining representative sequences and eliminating highly similar ones, which could lead to model overfitting. By clustering sequences at 60%, we ensure that our machine learning model is trained on a more diverse set of protein sequences, capturing a wider range of functional and structural variations. This approach helps to improve the model's generalizability while preventing the introduction of bias from overly similar sequences.
